# Field Bunds Planted with Sesame Conserve the Predatory Mirid Bug *Cyrtorhinus lividipennis* and Suppress Piercing-Sucking Rice Pests: Evidence from a Two-Year Field and Molecular Gut Content Analysis

**DOI:** 10.3390/insects17101066

**Published:** 2026-10-15

**Authors:** Lin Hong, Jie Wang, Xue-Wei Qi, Su Wang, Hong-Fan Chen, Jian-Hua Huang, Zheng-Xin Cheng, Zhong-Xian Lu, Feng Ge, Hong-Yi Wei, Yu-Yong Liang

**Affiliations:** 1 College of Agronomy, Jiangxi Agricultural University, Nanchang 330045, China; jxsnkyhl@163.com; 2 Institute of Plant Protection, Jiangxi Academy of Agricultural Sciences, Nanchang 330200, China; qixuewei77@163.com (X.-W.Q.); hongfan211@sohu.com (H.-F.C.); lyachjh@163.com (J.-H.H.); chengzxbeetle@163.com (Z.-X.C.); 3 Zhejiang Engineering Research Center for Biological Control of Crop Pathogens and Insects, Zhejiang Key Laboratory of Biology and Ecological Regulation of Crop Pathogens and Insects, Ministry of Agriculture and Rural Affairs Key Laboratory of Molecular Biology of Crop Pathogens and Insect Pests, State Key Laboratory of Rice Biology and Breeding, Institute of Insect Sciences, College of Agriculture and Biotechnology, Zhejiang University, Hangzhou 310058, China; wj_insect@126.com; 4 Institute of Plant Protection, Beijing Academy of Agriculture and Forestry Sciences, Beijing 100097, China; anthocoridae@163.com; 5 Institute of Plant Protection and Microbiology, Zhejiang Academy of Agricultural Sciences, Hangzhou 310021, China; luzxmh@163.com; 6 Jinan Engineering Research Center of Ecological Agricultural Products R&D and Industrialization, Institute of Plant Protection, Shandong Academy of Agricultural Sciences, Jinan 250131, China; gef@ioz.ac.cn

**Keywords:** biodiversity, sustainable agriculture, ecological engineering, functional plant, molecular gut-content analysis

## Abstract

Habitat management using flowering plants on rice bunds and field ridges surrounding rice paddies can enhance conservation biological control by supporting beneficial predatory insects. However, multi-year field evidence for sesame (*Sesamum indicum*) when deployed as these bund-side flowering strips, and there is no molecular gut-content confirmation that the key predatory mirid bug *Cyrtorhinus lividipennis* preys upon rice planthoppers pests under field conditions. Over a two-year field experiment, we compared rice paddies with sesame-planted bunds against control paddies without bunds. We surveyed major piercing-sucking pests (brown planthoppers *Nilaparvata lugens*, white-backed planthoppers *Sogatella furcifera*, leafhoppers, and thrips) and their primary predator *C. lividipennis* throughout different rice phenological growth stages. PCR-based laboratory digestion assays were used to quantify the detectability of *N. lugens* DNA in the gut of laboratory-reared and field-collected *C. lividipennis*. Sesame-planted bunds significantly increased natural-enemy abundance (*C. lividipennis*) and reduced planthoppers, leafhopper, and thrips densities. Treatment effects varied by rice stage, with suppression mainly occurring from booting/heading to grain filling and no significant differences at tillering, and effects at maturity were weak. In the laboratory, *N. lugens* DNA detection declined from 68.75% at 0 h to 0% at 16 h after feeding. In the field, 37.5% of *C. lividipennis* from sesame-bund paddies tested positive for *N. lugens* DNA. These findings provide field and molecular evidence that sesame-planted bunds conserve *C. lividipennis* and contribute to suppression of piercing-sucking rice pests, supporting their use as an auxiliary habitat-management tactic in rice integrated pest management.

## Simple Summary

Rice production suffers heavy damage from piercing-sucking pests such as planthoppers. Planting flowering functional plants along rice field bunds is an eco-friendly strategy to boost natural enemy insects and cut pesticide use. Sesame can supply nectar and pollen for beneficial insects, but we still lack multi-year field data and direct proof whether the key predatory mirid bug *Cyrtorhinus lividipennis* preys on brown planthoppers in sesame-enhanced rice fields. We set up two-year field trials comparing rice fields with sesame-planted bunds and ordinary control fields. We also ran laboratory DNA degradation tests and molecular gut-content PCR to verify predation. Our results showed that bund-grown sesame increased populations of the predatory mirid and suppressed rice pests, with stronger effects during mid-late rice growth stages. Molecular tests confirmed field predation by *C. lividipennis* on brown planthoppers. This study supports using sesame strips as an auxiliary pest-control measure within rice eco-friendly integrated pest management.

## 1. Introduction

Rice is a staple food for more than half of the global population, and its sustainable production is central to food security. However, rice yields and quality are persistently constrained by insect pests, especially sap-feeding planthoppers, leafhoppers, and thrips, as well as lepidopteran stem borers and leaf folders [1,2,3]. These pests frequently cause substantial yield losses and destabilize rice agroecosystems across major rice-growing regions. For decades, rice pest management has heavily relied on synthetic insecticides, which has triggered a suite of adverse ecological consequences, accelerated pest resistance, mortality of non-target natural enemies, biodiversity erosion within farmland, and widespread environmental contamination [4,5]. Natural enemies play crucial ecological and economic roles in rice pest management, serving as key biological agents for achieving green pest management and sustainable agriculture [4,6,7,8,9]. Against this background, habitat manipulation, a core component of integrated pest management (IPM), has received increasing attention [10,11,12,13]. As one key tactic of conservation biological control, this strategy modifies field habitats by establishing functional plant strips to conserve natural enemy populations, strengthen natural pest suppression services, and reduce dependency on chemical inputs [10,14,15,16,17].

Rice field bunds and adjacent non-crop habitats are important semi-natural components of paddy ecosystems [18,19]. They can provide shelter, nectar, pollen, alternative prey, and overwintering sites for predators and parasitoids, thereby supporting natural enemy conservation and pest suppression [20,21,22,23]. Flowering plants established on rice bunds have been widely used in ecological engineering, and several plant species can increase natural enemy abundance and reduce pest populations [24,25,26,27,28,29]. Sesame [*Sesamum indicum* L. (Pedaliaceae)] is a particularly promising functional plant because its flowers provide accessible nectar and pollen to multiple natural enemies without strongly benefiting key rice pests [30,31,32]. Previous studies have shown that sesame can enhance the performance of the predatory mirid bug, *Cyrtorhinus lividipennis* Reuter (Hemiptera: Miridae), improve parasitoid longevity and fecundity, and increase parasitism of planthopper eggs [27,31,33]. Sesame has also been used as a banker plant for zoophytophagous predators such as *Nesidiocoris tenuis* (Reuter) (Hemiptera: Miridae) [34,35,36,37]. Nevertheless, most evidence comes from short-term or single-stage studies, and the multi-year field effects of sesame planted on rice bunds on rice planthoppers, leafhoppers, thrips, and their key predator *C. lividipennis* remain insufficiently documented.

The mirid bug *C. lividipennis* is a dominant in-field predator of rice planthoppers [i.e., brown planthoppers *Nilaparvata lugens* (Stål) (Hemiptera: Delphacidae), and white-backed planthoppers *Sogatella furcifera* (Stål) (Hemiptera: Delphacidae)] and contributes substantially to natural biological control in paddy fields, especially because it preys on planthopper eggs and nymphs [31,38,39]. Conventional field population surveys can only reveal temporal synchrony between predator and pest abundances but cannot directly verify actual predation events occurring under field conditions [39,40]. Molecular predator gut-content analysis provides a powerful tool for identifying predator-prey trophic links, especially for small or cryptic arthropods [40,41]. Multiplex real-time PCR assays have been developed to detect DNA of multiple rice planthoppers in predator guts, including *N. lugens* [39]. Because prey DNA degrades after ingestion, laboratory measurements of DNA detectability over digestion time are essential for interpreting field positive rates [39,40]. Yet direct molecular evidence that *C. lividipennis* preys on *N. lugens* in rice fields with sesame-planted bunds is still lacking.

To address these gaps, this study combined a two-year field experiment with molecular predator gut-content analysis. The specific objectives were to (1) compare the population dynamics of brown planthoppers (*N. lugens*), white-backed planthoppers (*S. furcifera*), leafhoppers, thrips, and *C. lividipennis* between rice paddies with sesame-planted bunds and control paddies; (2) determine the post-feeding digestion dynamics of *N. lugens* prey DNA in *C. lividipennis* under laboratory conditions; and (3) detect *N. lugens* prey DNA in field-collected *C. lividipennis* to provide direct molecular evidence of in-field predation. This work provides multi-year field demographic data together with molecular trophic evidence for the application of bund-planted sesame as a functional plant and delivers empirical support for habitat-manipulation-based IPM strategies in rice production.

## 2. Materials and Methods

### 2.1. Experimental Site and Plant Materials

Field experiments were carried out from June to October in both 2023 and 2024 at Banlian Village, Bayi Township, Nanchang County, Nanchang City, Jiangxi Province, China (28°32′ N, 115°57′ E). The rice cultivar Taiyou 398 (Jiangxi Modern Seed Industry, Nanchang, China) and sesame cultivar Ganzhi-9 (Jiangxi Academy of Agricultural Sciences, Nanchang, China) were used in this study. In 2023, rice seedlings were transplanted on July 27 and harvested in late October; sesame was sown on August 8 and harvested in late October. In 2024, rice was direct-seeded on July 9 and harvested in late October, while sesame was sown on July 20 and harvested in early October.

Two treatments were established in independent field plots: rice fields with sesame planted along surrounding field bunds (treatment) and conventional rice fields without sesame intercropping on bunds (control). Each treatment contained three replicate plots. Each experimental plot covered a rectangular area of 900 m^2^ (25 m × 36 m). Spacing of more than 50 m was maintained between replicate plots within the same treatment, as well as between adjacent plots of different treatments, to mitigate edge effects and insect dispersal interference. All plots were managed following local conventional agronomic practices, with no chemical insecticides or pest-control agents applied. The width of each field bund was 70 cm, and the sesame strip was 50 cm in width. Sesame was sown along the full-perimeter bunds of each rectangular plot, giving a total sesame-strip length of 122 m per plot.

### 2.2. Field Survey of Dominant Arthropods

Arthropod surveys commenced at the rice tillering stage and continued until one week prior to rice harvest in 2023 and 2024. We monitored population dynamics of rice pests including rice planthoppers, rice leafhoppers and thrips, together with their natural predator mirid bug, *C. lividipennis*. Each experimental rice plot was divided into 20 sampling points arranged in a 4 × 5 grid. Sampling points were evenly distributed across the inner area of each plot, with the outermost sampling points situated 5–6 m from the plot perimeter (field bunds). This buffer distance avoided sampling in the immediate border zone adjacent to bunds and reduced potential edge-effect bias driven by arthropod immigration from bund habitats. At each sampling point, the beat-plate method was adopted: a white rectangular plastic tray (50 cm long × 30 cm wide × 6 cm high) was used to sample two rice hills. The number of pests and natural enemies in each tray was counted rapidly.

Brown planthoppers and white-backed planthoppers were distinguished directly in the field according to diagnostic morphological traits. Leafhoppers [predominantly *Nephotettix* spp. (Hemiptera: Cicadellidae)] and thrips [predominantly *Stenchaetothrips biformis* (Bagnall) (Thysanoptera: Thripidae)] were pooled as functional groups, because species-level identification could not be completed during rapid field counts. The predatory mirid bug *C. lividipennis* was preliminarily identified in the field by its distinctive morphology; representative specimens were subsequently confirmed in the laboratory under a stereomicroscope using standard taxonomic keys to differentiate it from other co-occurring Miridae. The 20 subsampling points within each plot were averaged to produce one mean value per plot for each sampling date. Field counts were converted to population density per 100 rice hills:

N=∑n∗2.5

where *N* was the converted density (ind. per 100 hills), and *n* was the insect count at each sampling point (ind. per two hills).

Individual sampling points represented subsamples rather than independent replicates. Surveys were performed approximately every seven days; sampling dates were adjusted by one day in advance or delayed under rainy weather conditions.

### 2.3. Molecular Assays for Predation of Cyrtorhinus lividipennis on Nilaparvata lugens

Experimental individuals of both the predatory mirid bug *C. lividipennis* and brown planthoppers were originally field-collected from rice paddies in July 2025 from the experimental field of the Institute of Plant Protection, Jiangxi Academy of Agricultural Sciences, Jiangxi Province, China (115°56′59″ N, 28°32′30″ E). Brown planthoppers were reared on rice plants of cultivar Taiyou 398 (Jiangxi modern seed industry, Nanchang, China), while *C. lividipennis* was maintained by feeding brown planthopper nymphs. Both species were continuously mass-reared for three successive generations in insect cages (50 cm × 50 cm × 50 cm) prior to bioassays. All colonies were maintained under laboratory conditions (26 ± 1 °C, 70 ± 5% RH, and a 16 L:8 D photoperiod). Newly emerged *C. lividipennis* adults (2–3 days post-eclosion; 200 individuals in total, 20 adults for each post-feeding time point) were individually placed in 3.5 cm Petri dishes and starved for 24 h. Each starved adult was provided with one second-instar *N. lugens* nymph as prey. The moment when predation was completed was defined as *t* = 0, and individuals that failed to finish consuming prey within 2 h were discarded. Predators were collected at 0, 1, 2, 4, 6, 8, and 16 h post-feeding, and naturally dead individuals were excluded. Collected specimens were preserved in absolute ethanol and stored at −20 °C. Total genomic DNA was extracted from individual *C. lividipennis* using a TIANamp Micro DNA Kit (Tiangen Biotech, Beijing, China) following the manufacturer’s instructions. Species-specific primers for *N. lugens* were adopted from Wang et al. [42]: BPH-F: TTGATTTCGAGGTTCGTCTGTATG and BPH-R: ACATGCACAAATCACCACAGAGT. The polymerase chain reaction (PCR) thermal-cycling profile for *N. lugens*-specific primer pair was as follows: an initial denaturation step at 95 °C for 5 min, followed by 35 cycles consisting of denaturation at 94 °C for 15 s, annealing at 55 °C for 30 s, and extension at 72 °C for 1 min. A final extension was performed at 72 °C for 5 min, and reactions were held at 4 °C indefinitely upon completion. PCR was applied to detect prey DNA, and positive detection rates of *N. lugens* in predator guts across different digestion durations were calculated [39].

Field-collected *C. lividipennis* individuals were immediately immersed in absolute ethanol upon sampling, transported to the laboratory, and stored at −20 °C. The positive prey DNA detection rate for field samples was determined using the same protocol as for laboratory specimens.

### 2.4. Statistical Analysis

All statistical analyses were conducted in R software (version 4.3.1) [43]. The experimental unit was the plot, with three replicate plots per treatment. All statistical analyses were performed on plot-level mean values, not individual subsample data, to avoid pseudoreplication. Generalized linear mixed-effects models (GLMMs) with a negative binomial distribution were fitted via the ‘glmmTMB’ function from the glmmTMB package [44] to assess the effects of sesame intercropping on the abundance of rice planthoppers, the key predator *C. lividipennis*, and other pests (thrips and leafhoppers). Treatment (sesame-intercropped bunds versus control), rice developmental stage (tillering, booting, heading, grain-filling and maturity), and their interaction were included as fixed effects. Given substantial inter-annual environmental variation, including differences in weather conditions, data collected in 2023 and 2024 were analyzed separately. Year was not included as either a fixed or random effect in the GLMMs. Plot identity (ID) was incorporated as a random intercept to account for temporal autocorrelation derived from repeated sampling within each experimental plot. All GLMMs achieved successful convergence without singular-fit warnings. Plot ID was specified as a random intercept to account for repeated sampling over time within each plot. We acknowledge that the random effect had a relatively small number of levels (*n* = 3 replicate plots per treatment), which may limit the precision of variance component estimates.

Significance of fixed factors was tested via Type II Wald *χ*^2^ tests using the ‘Anova’ function in the car package [45]. Where significant interaction terms occurred, separate sub-models were constructed to evaluate treatment effects for each individual rice growth stage. Model diagnostics, including overdispersion, were assessed using the ‘testDispersion’ function from the R package DHARMa (version 0.5.0), with this ratio compared against chi-square residuals. Graphical visualizations were generated using GraphPad Prism 9.0.

## 3. Results

### 3.1. Effects of Sesame-Planted Field Bunds on Population Dynamics of Rice Planthoppers

**Figure 1:**
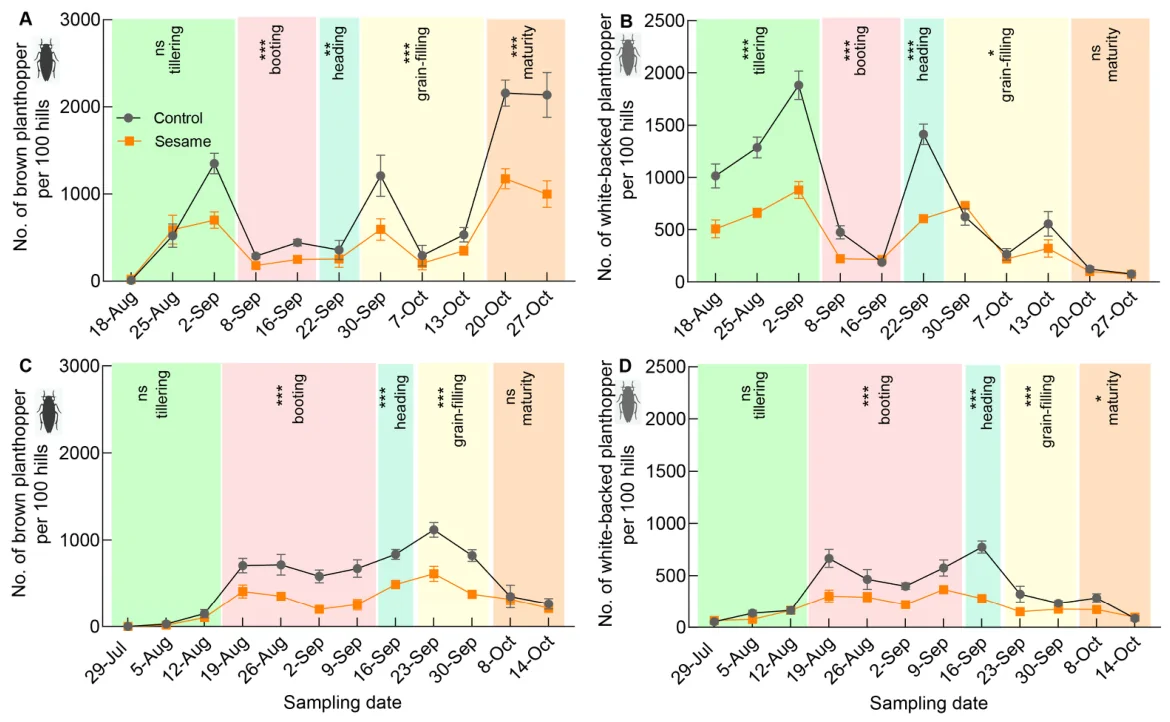
Mean (±SE) abundance of brown planthoppers, *Nilaparvata lugens* (**A**,**C**), and white-backed planthoppers, *Sogatella furcifera* (**B**,**D**), in control and sesame-intercropped bund fields in 2023 (**A**,**B**) and 2024 (**C**,**D**). Shaded background indicates rice tillering, booting, heading, grain-filling, and maturity stages, respectively. ns *p* > 0.05, * *p* < 0.05, ** *p* < 0.01, *** *p* < 0.001.

Cumulative total captures of focal piercing-sucking pests across the two-year sampling period are presented in Table S1. In 2023, total captures of brown planthoppers were 11,162 (control) and 6384 (sesame bunds), and white-backed planthoppers were 9483 (control) and 5443 (sesame bunds). In 2024, total captures were 4996 (control) and 2845 (sesame bunds) for white-backed planthoppers, and 7494 (control) and 4017 (sesame bunds) for brown planthoppers. In both 2023 and 2024, the densities of brown planthoppers and white-backed planthoppers were significantly affected by sesame planted on field bunds, rice phenological stage, and their interaction (Figure 1). Brown planthoppers abundance was significantly influenced by treatment, rice phenology, and their interaction in both years (2023: Treatment, *df* = 1; *χ*^2^ = 135.40; *p* < 0.001; Rice stage, *df* = 4; *χ*^2^ = 1260.85; *p* < 0.001; Treatment: Rice stage, *df* = 4; *χ*^2^ = 24.34; *p* < 0.001; 2024: Treatment, *df* = 1; *χ*^2^ = 438.09; *p* < 0.001; Rice stage, *df* = 4; *χ*^2^ = 1363.79; *p* < 0.001; Treatment: Rice stage, *df* = 4; *χ*^2^ = 63.19; *p* < 0.001). For white-backed planthoppers: treatment, rice stage, and the treatment × rice-stage interaction all exerted significant effects in 2023 (Treatment: *df* = 1; *χ*^2^ = 336.04; *p* < 0.001; Rice stage: *df* = 4; *χ*^2^ = 1808.50; *p* < 0.001; Treatment: Rice stage: *df* = 4; *χ*^2^ = 85.17; *p* < 0.001) and 2024 (Treatment: *df* = 1; *χ*^2^ = 326.03; *p* < 0.001; Rice stage: *df* = 4; *χ*^2^ = 1212.60; *p* < 0.001; Treatment: Rice stage: *df* = 4; *χ*^2^ = 63.95; *p* < 0.001).

Separate stage-wise analyses revealed that sesame-planted bunds significantly reduced the population densities of both planthoppers species mainly from the booting/heading stage through grain filling in both sampling years. By contrast, treatment effects were inconsistent at the tillering stage: only white-backed planthoppers showed a significant treatment response in 2023, whereas no significant effects were detected for either planthoppers species at tillering in 2024 (Table 1).

### 3.2. Effects of Sesame-Planted Field Bunds on Population Dynamics of the Planthopper Predator Cyrtorhinus lividipennis

**Figure 2:**
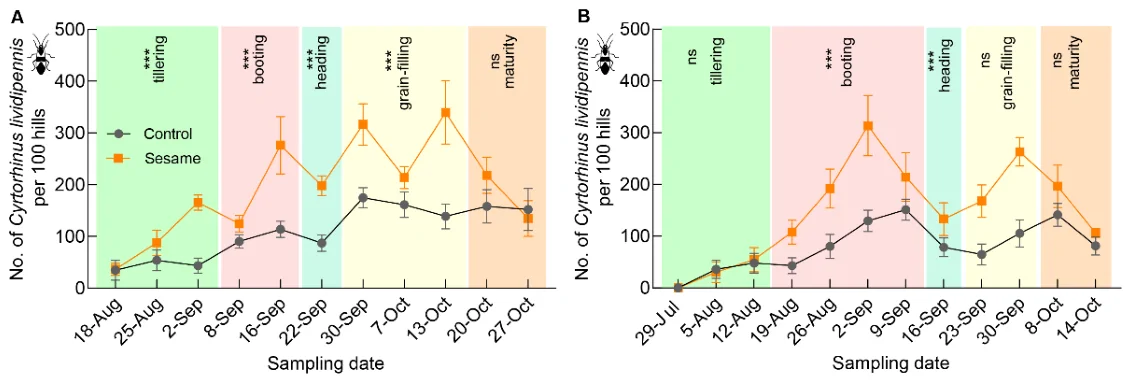
Mean (±SE) abundance of the predatory mirid bug *Cyrtorhinus lividipennis* in control and sesame-intercropped bund fields in 2023 (**A**) and 2024 (**B**). Shaded background indicates rice tillering, booting, heading, grain-filling, and maturity stages, respectively. ns *p* > 0.05, *** *p* < 0.001.

Total cumulative captures of the predatory mirid bug *C. lividipennis* over the two-year survey are provided in Table S1. In 2023, 2042 individuals were collected from control plots and 2845 from sesame-bund plots; in 2024, 1246 individuals were captured in control plots versus 2042 in sesame-bund plots. Both in 2023 and 2024, sesame-planted field bunds significantly increased the abundance of the predatory mirid bug *C. lividipennis*, alongside significant effects of rice phenological stage and the treatment-by-phenology interaction (Figure 2). In 2023, *C. lividipennis* density differed significantly by treatment (*df* = 1; *χ*^2^ = 178.55; *p* < 0.001), rice stage (*df* = 4; *χ*^2^ = 367.86; *p* < 0.001), and the treatment × rice-stage interaction (*df* = 4; *χ*^2^ = 30.04; *p* < 0.001). In 2024, significant effects were detected for treatment (*df* = 1; *χ*^2^ = 147.64; *p* < 0.001), rice stage (*df* = 4; *χ*^2^ = 319.71; *p* < 0.001), and the treatment-stage interaction (*df* = 4; *χ*^2^ = 30.12; *p* < 0.001). Stage-wise analyses revealed that sesame-planted bunds substantially boosted *C. lividipennis* populations across tillering to grain-filling stages in 2023, while significant population enhancement was restricted to booting and heading stages in 2024. At rice maturity, no significant treatment difference was detected for either planthoppers species in 2023. In 2024, a significant treatment effect was observed for brown planthoppers, while no significant difference was found for white-backed planthoppers (Table 1).

### 3.3. Effects of Sesame-Planted Field Bunds on Population Dynamics of Rice Leafhoppers and Thrips

**Figure 3:**
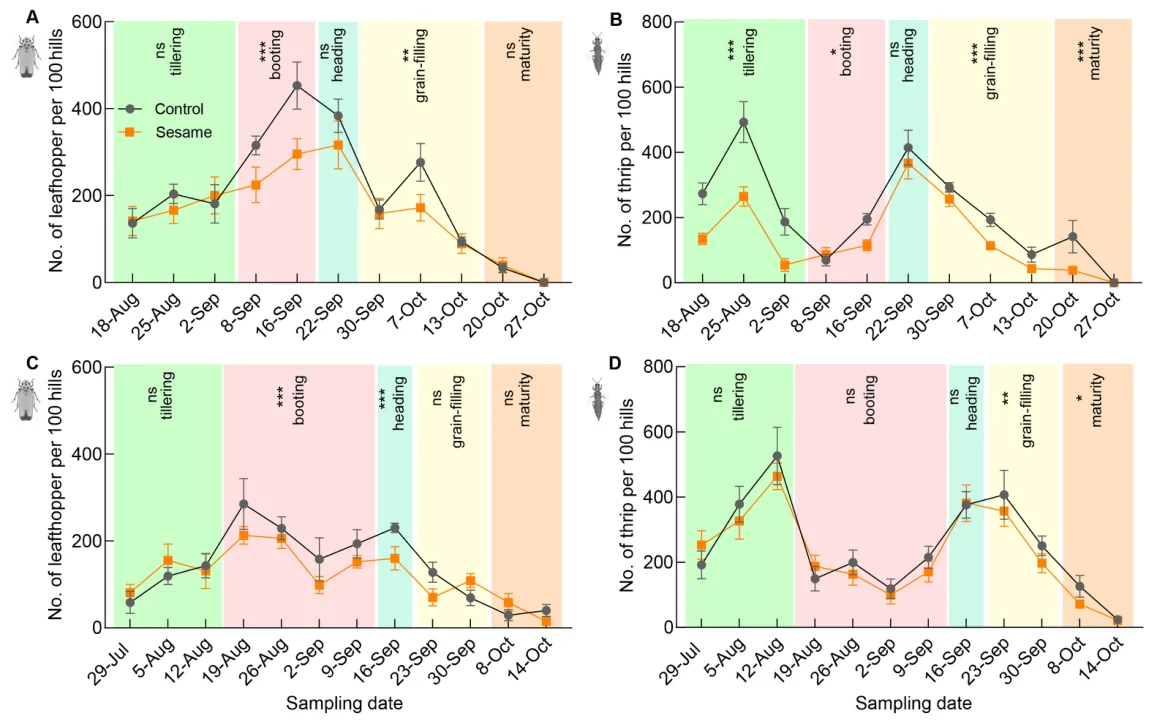
Mean (±SE) abundance of leafhoppers (**A**,**C**) and thrips (**B**,**D**) in control and sesame-intercropped bund fields in 2023 (**A**,**B**) and 2024 (**C**,**D**). Shaded background indicates rice tillering, booting, heading, grain-filling, and maturity stages, respectively. ns *p* > 0.05, * *p* < 0.05, ** *p* < 0.01, *** *p* < 0.001.

Cumulative captures of leafhoppers and thrips across the two-year survey are provided in Table S1. Total leafhopper counts were 2690 (control) and 2160 (sesame bunds) in 2023, and 2017 (control) and 1786 (sesame bunds) in 2024. For thrips, total captures were 2809 (control) and 2176 (sesame bunds) in 2023, and 3551 (control) and 3229 (sesame bunds) in 2024. Both in 2023 and 2024, leafhopper densities were significantly affected by sesame-planted field bunds, rice phenological stage, and their interaction (Figure 3A,C). In 2023, leafhopper abundance differed significantly by treatment (*df* = 1; *χ*^2^ = 31.59; *p* < 0.001), rice stage (*df* = 4; *χ*^2^ = 760.12; *p* < 0.001), and their interaction (*df* = 4; *χ*^2^ = 15.37; *p* = 0.004). Similarly, in 2024, significant effects were detected for treatment (*df* = 1; *χ*^2^ = 12.32; *p* < 0.001), rice stage (*df* = 4; *χ*^2^ = 433.52; *p* < 0.001), and the treatment–stage interaction (*df* = 4; *χ*^2^ = 17.97; *p* = 0.001). Stage-wise analyses revealed that sesame-planted bunds reduced leafhopper populations mainly at the booting and heading stages in both years, while no treatment differences were observed at tillering, grain-filling, or maturity stages (Table 1). Thrips abundance was significantly affected by sesame bund planting and rice phenology in 2023 (Figure 3B), together with a significant treatment × rice-stage interaction (Treatment: *df* = 1; *χ*^2^ = 104.59; *p* < 0.001; Rice stage: *df* = 4; *χ*^2^ = 637.24; *p* < 0.001; Treatment: Rice stage: *df* = 4; *χ*^2^ = 45.27; *p* < 0.001). In 2024, thrips density responded significantly to treatment and rice developmental stage (Figure 3D), but their interaction was not statistically significant (Treatment: *df* = 1; *χ*^2^ = 6.69; *p* = 0.010; Rice stage: *df* = 4; *χ*^2^ = 763.90; *p* < 0.001; Treatment: Rice stage: *df* = 4; *χ*^2^ = 6.97; *p* = 0.138). Stage-specific comparisons indicated that sesame-planted bunds suppressed thrips populations primarily at booting and grain-filling stages in 2023, whereas the treatment effect in 2024 was consistent across rice phenological stages without pronounced stage-dependent variation (Table 1).

### 3.4. Molecular Assays of Predation by Cyrtorhinus lividipennis on Nilaparvata lugens Under Field Conditions

**Figure 4:**
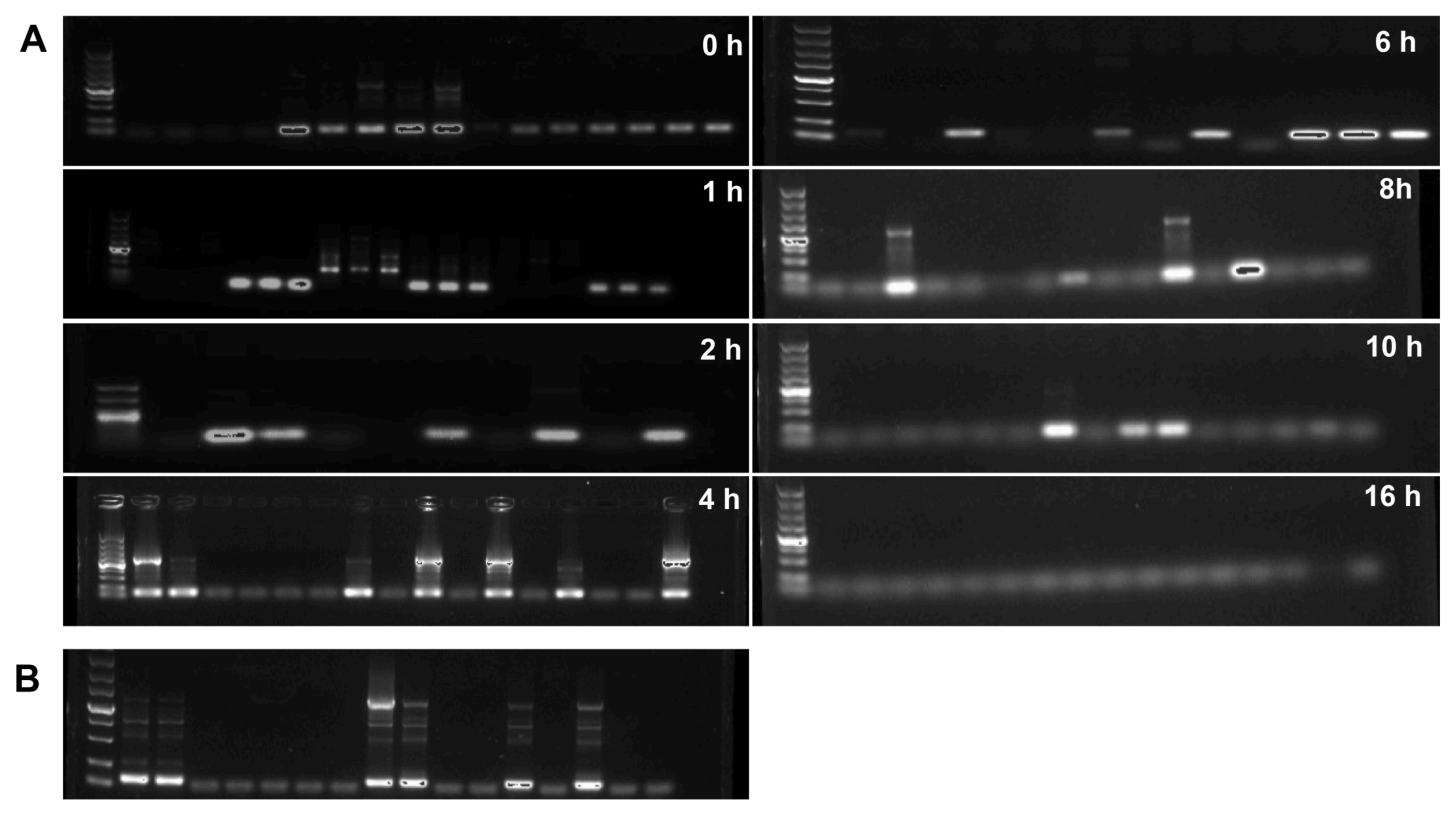
PCR-amplified products of *Nilaparvata lugens* DNA detected in *Cyrtorhinus lividipennis* adult gut contents. (**A**) Electrophoretic profiles from laboratory digestion assays across a time series (0–16 h post-predation). (**B**) Electrophoretic results for field-sampled *C. lividipennis*.

In the laboratory feeding experiment, the positive detection rate of *N. lugens* prey DNA within *C. lividipennis* guts declined progressively with increasing post-feeding digestion time (Figure 4). The prey DNA positive rate was 68.75% (11/16) at 0 h after feeding, 56.25% (9/16) at 1 h, 50.00% (5/10) at 2 h, 43.75% (7/16) at 4 h, 41.67% (5/12) at 6 h, 25.00% (4/16) at 8 h, 18.75% (3/16) at 10 h, and dropped to 0% (0/16) at 16 h post-feeding, indicating that prey DNA of *N. lugens* could no longer be amplified from predator gut contents after 16 h of digestion. Under field conditions, the field-collected *C. lividipennis* individuals yielded a *N. lugens* prey-DNA positive detection rate of 37.50% (6/16), providing molecular evidence for in situ predation of *C. lividipennis* on *N. lugens* in rice paddies with sesame-planted bunds.

## 4. Discussion

Two consecutive years of field experiments demonstrated that planting sesame along rice field bunds significantly increased field abundances of the predatory mirid bug *C. lividipennis* while concurrently suppressing populations of rice planthoppers, leafhoppers, and thrips. Significant treatment-by-rice-phenology interactions were consistently detected across both experimental years, indicating that pest-suppressive and predator-boosting effects of sesame bund strips were strongly dependent on rice growth stages rather than being uniformly effective throughout the entire growing season. Complementary molecular assays provided supplementary trophic information: laboratory feeding experiments documented the time-dependent degradation pattern of *N. lugens* prey DNA within *C. lividipennis* digestive tracts, and gut-content PCR of field-collected predators yielded direct molecular confirmation of field predation on brown planthoppers. Collectively, our results highlight that bund-planted sesame exerts stage-dependent biological control services in paddy ecosystems.

The suppressive effects of sesame-planted bunds on rice planthoppers are consistent with previous reports that nectar-producing plants and habitat diversification can reduce rice planthopper populations by enhancing natural enemies [18,24,26,27]. In rice ecological engineering, sesame has been shown to benefit *C. lividipennis* and parasitoids of planthoppers, which can translate into stronger biological control [27,30,31]. The stronger treatment effects from booting/heading to grain filling likely reflect the phenological matching between pest population growth and natural enemy establishment. During early tillering, planthoppers densities are often relatively low, and generalist predators may still be colonizing the crop or relying on alternative prey [20,46]. As rice enters the booting and heading stages, planthoppers populations increase rapidly, and the predator populations supported by sesame nectar and pollen can exert stronger top-down control [27,31]. This stage-dependent pattern is also consistent with the idea that habitat management effects are not uniform throughout the cropping season but depend on crop phenology and natural enemy population build-up [14,21].

The increased abundance of *C. lividipennis* in sesame-bund plots is one of the key findings of this study. *Cyrtorhinus lividipennis* is a major predator of rice planthopper eggs and nymphs, and its conservation can contribute substantially to planthoppers regulation [31,38]. Sesame flowers provide nectar and pollen, which can improve predator survival, longevity, fecundity, and predation capacity [31,34,35,36,47]. In addition, rice bunds planted with sesame can serve as refuge habitats that support predator establishment and movement into the crop [20,26,27]. The population enhancement of *C. lividipennis* was concentrated in the middle and late rice stages, while no treatment difference occurred at maturity. This pattern may be related to rice plant nutritional quality, prey availability, and the flowering phenology of sesame. It may also reflect seasonal migration and dispersal dynamics, because *C. lividipennis* can co-migrate with planthoppers across regions [33]. Thus, sesame-planted bunds appear to strengthen predator populations when planthopper pressure is increasing, but the effect weakens as the rice crop matures.

Leafhopper suppression in sesame-bund plots is also broadly consistent with previous work showing that vegetation strips and diversified rice landscapes can reduce leafhopper abundance, sometimes additively with varietal resistance [20]. For thrips, however, the treatment with stage interaction was significant in 2023 but not in 2024. This indicates some year-to-year variation in the strength and stage specificity of sesame effects. Such variation may arise from differences in initial pest density, weather conditions, rice growth, or the local phenology of natural enemy communities. Therefore, the thrips results should be interpreted cautiously: sesame-planted bunds may contribute to thrips suppression, but the effect is not equally strong across years or stages. More generally, the present results do not suggest that sesame-planted bunds alone can fully suppress rice pests. Rather, they support the view that habitat management should be integrated with other IPM tactics, such as resistant varieties, rational fertilization, and reduced-risk pesticides, to achieve robust and sustainable pest control [2,20,48].

The molecular results provide an important complement to the population data. The laboratory digestion experiment showed that *N. lugens* prey DNA could be detected in *C. lividipennis* guts shortly after feeding but declined rapidly and became undetectable by 16 h. This is consistent with the general principle that prey DNA detectability is time-limited and must be interpreted within a digestion window [39,40]. The field detection rate of 37.5% demonstrates that *C. lividipennis* indeed consumed *N. lugens* in rice paddies with sesame-planted bunds. This is direct trophic evidence that complements previous reports based on population correlation or laboratory predation assays [31,39]. However, the positive detection rate should not be converted directly into predation rate or prey consumption. A positive PCR result only indicates that prey DNA was present in the gut at sampling; it does not indicate how many prey individuals were consumed, whether the predation event occurred recently, or whether the predator also fed on other prey. The detection probability is influenced by digestion time, meal size, temperature, primer sensitivity, sampling interval, and the availability of alternative prey [39,40]. The relatively limited sample size in this study further cautions against quantitative extrapolation. Therefore, the molecular evidence should be interpreted as qualitative confirmation of in-field predation, rather than a precise measure of predation intensity.

The pest-suppressive effect observed here is most likely driven by enhanced predator abundance, especially the conservation of *C. lividipennis* through sesame-provided floral resources and bund habitat. This is supported by the simultaneous increase in *C. lividipennis* and decrease in planthoppers populations, and by the molecular detection of *N. lugens* DNA in field-collected predators. Similar predator-mediated mechanisms have been reported for sesame and other flowering plants in rice ecosystems [18,26,27,31]. However, we cannot rule out potential behavioral effects of sesame volatile compounds on herbivores. Some plant extracts and volatile compounds can repel rice planthoppers while attracting *C. lividipennis* [49,50], and herbivore-induced plant volatiles can mediate natural enemy foraging [38,51]. Whether sesame itself releases behaviorally active volatiles that directly interfere with planthopper host location or reproduction was not tested in this study. Thus, a direct repellent or volatile-mediated mechanism remains a possible secondary explanation, but further behavioral and chemical ecology experiments are required before any conclusion can be drawn.

Several limitations should be acknowledged. First, the experimental fields were not treated with chemical insecticides. In real rice production, farmers often apply insecticides, which can kill natural enemies and weaken the benefits of habitat management [5,27,38,51]. Therefore, the effectiveness of sesame-planted bunds in conventionally sprayed fields may differ from that observed here. Second, the molecular analysis had a limited sample size and could provide only qualitative evidence of predation because prey DNA degrades within hours and positive rates cannot be equated with predation rates [39,40]. Additionally, in this field survey, leafhoppers and thrips were recorded as pooled functional groups rather than resolved to species level. This grouping was necessary given the constraints of rapid in-field counting, and regional surveys indicate that assemblages were dominated by *Nephotettix* spp. (leafhoppers) and *S. biformis* (thrips). Finally, only one sesame-bund configuration was evaluated. Bund width, sesame strip width, sowing date, and spatial arrangement can influence natural enemy movement and pest suppression [20,21,25,52]. The gradient effects of these design parameters were not examined.

## 5. Conclusions

From an applied perspective, planting sesame on rice field bunds is a low-cost and environmentally compatible habitat-management measure that can enhance *C. lividipennis* populations and contribute to the suppression of planthoppers, leafhoppers, and thrips. It can therefore be recommended as an auxiliary tool in rice IPM programs, particularly in ecological engineering schemes that aim to reduce pesticide dependence [18,23,53,54,55]. However, it should not be viewed as a substitute for other management tactics. Rather, it should be integrated with resistant varieties, rational water and nutrient management, and judicious pesticide use to achieve stable pest control [1,2,20]. Future research should prioritize several directions. First, multi-site and multi-year trials should verify regional applicability under diverse agroecological conditions. Second, behavioral and chemical ecology studies should examine whether sesame volatiles, nectar, or pollen directly affect *C. lividipennis* and pest behavior. Third, field trials should evaluate the performance of sesame-planted bunds under realistic pesticide-use scenarios, because pesticide applications may alter natural enemy populations and biological control outcomes [5,27]. Finally, future work should consider landscape context, because the effects of flower strips and habitat management can vary with landscape complexity and the composition of surrounding non-crop habitats [13,56,57,58,59]. Addressing these questions will help translate sesame-bund habitat management into a more reliable and scalable strategy for sustainable rice pest management.

## Figures and Tables

**Table 1:** Effects of treatment (sesame intercrop vs. control) on the abundance of piercing-sucking rice pests and predatory mirid bug *Cyrtorhinus lividipennis* at the different rice phenological stages in 2023 and 2024. Estimates, degrees of freedom, *χ*^2^-values, and *p*-values are retrieved from generalized linear mixed models tested with a Wald chi-square test.

			2023				2024		
		Est.	*df*	*χ* ^2^	*p*	Est.	*df*	*χ* ^2^	*p*
Brown planthoppers									
	Tillering	−0.03	1	0.06	0.799	−0.30	1	3.4	0.065
	Booting	−0.54	1	53.9	<0.001 ***	−0.81	1	325.1	<0.001 ***
	Heading	−0.34	1	8.7	0.003 **	−0.54	1	52.8	<0.001 ***
	Grain filling	−0.41	1	32.2	<0.001 ***	−0.67	1	146.6	<0.001 ***
	Maturity	−0.65	1	184.2	<0.001 ***	−0.13	1	2.8	0.095
White-backed planthoppers									
	Tillering	−0.67	1	195.1	<0.001 ***	−0.15	1	2.8	0.097
	Booting	−0.40	1	26.8	<0.001 ***	−0.57	1	184.1	<0.001 ***
	Heading	−0.83	1	168.3	<0.001 ***	−1.02	1	189.1	<0.001 ***
	Grain filling	−0.16	1	6.2	0.013 *	−0.52	1	49.1	<0.001 ***
	Maturity	−0.14	1	2.3	0.133	−0.25	1	6.5	0.011 *
Leafhoppers									
	Tillering	−0.01	1	0.03	0.874	0.13	1	2.1	0.146
	Booting	−0.38	1	36.1	<0.001 ***	−0.24	1	17.1	<0.001 ***
	Heading	−0.17	1	3.5	0.060	−0.37	1	13.8	<0.001 ***
	Grain filling	−0.24	1	9.9	0.002 **	−0.08	1	0.4	0.522
	Maturity	0.06	1	0.06	0.809	0.05	1	0.08	0.773
Thrips									
	Tillering	−0.72	1	90.4	<0.001 ***	−0.02	1	0.12	0.727
	Booting	−0.22	1	4.7	0.030 *	−0.09	1	1.74	0.187
	Heading	−0.12	1	3.4	0.065	0.01	1	0.01	0.911
	Grain filling	−0.34	1	16.6	<0.001 ***	−0.18	1	7.0	0.008 **
	Maturity	−0.92	1	17.2	<0.001 ***	−0.37	1	6.0	0.014 *
Predatory mirid bug *Cyrtorhinus lividipennis*									
	Tillering	0.78	1	54.1	<0.001 ***	−0.03	1	0.03	0.870
	Booting	0.59	1	43.2	<0.001 ***	0.68	1	80.0	<0.001 ***
	Heading	0.78	1	32.2	<0.001 ***	0.52	1	14.6	<0.001 ***
	Grain filling	0.60	1	96.0	<0.001 ***	0.90	1	78.8	<0.001 ***
	Maturity	0.12	1	1.6	0.209	0.29	1	7.5	0.006 **

Note: * *p* < 0.05, ** *p* < 0.01, *** *p* < 0.001.

## Data Availability

The original contributions presented in this study are included in the article. Further inquiries can be directed to the corresponding authors.

## Supplementary Materials

The following supporting information can be downloaded at: https://www.mdpi.com/article/10.3390/insects17101066/s1, Table S1: Total number of insect individuals (individual/100 hills) sampled across treatments and years during the two-year field survey. Total counts of brown planthoppers (*Nilaparvata lugens*), white-backed planthoppers (*Sogatella furcifera*), leafhoppers, thrips, and the predatory mirid bug *Cyrtorhinus lividipennis* collected from rice plots with sesame-planted bunds and control bunds in 2023 and 2024. Sampling was performed using the beat-plate method across different rice phenological stages.
