# Supplementary material for: Field Bunds Planted with Sesame Conserve the Predatory Mirid Bug Cyrtorhinus lividipennis and Suppress Piercing-Sucking Rice Pests: Evidence from a Two-Year Field and Molecular Gut Content Analysis
Source: Insects. 2026 Oct 15;17(10):1066. doi: 10.3390/insects17101066 (PMC13705345; doi:10.3390/insects17101066)
Supplement: Supplementary file 1 [file insects-17-01066-s001.zip › insects-4625734-supplementary.pdf]

TABLE S1. Total number of insect individuals (individual/100 hills) sampled across treatments and years during the two-year field survey. Total counts of brown planthopper (*Nilaparvata lugens*), white-backed planthopper (*Sogatella furcifera*), leafhoppers, thrips, and the predatory mirid bug *Cyrtorhinus lividipennis* collected from rice plots with sesame-planted bunds and control bunds in 2023 and 2024. Sampling was performed using the beat-plate method across different rice phenological stages.

**Treatment**	**Control**			**Sesame**			**Control**			**Sesame**		
	**Plot 1**	**Plot 2**	**Plot 3**	**Plot 1**	**Plot 2**	**Plot 3**	**Plot 1**	**Plot 2**	**Plot 3**	**Plot 1**	**Plot 2**	**Plot 3**
**2023**	**white-backed planthopper**						**brown planthopper**					
18-Aug	798	1188	1058	558	343	623	20	10	0	25	13	30
25-Aug	1125	1468	1268	678	578	728	283	538	748	908	353	513
2-Sep	1633	1913	2100	988	723	928	1360	1550	1143	848	528	728
8-Sep	350	548	528	193	260	213	238	303	323	183	143	213
16-Sep	188	130	250	268	198	180	388	428	513	303	213	233
22-Sep	1253	1590	1393	668	538	610	568	198	310	183	443	135
30-Sep	768	608	493	708	768	720	1628	1193	808	373	798	610
7-Oct	353	163	280	188	265	208	528	160	193	113	358	148
13-Oct	378	778	513	268	213	483	388	673	538	280	323	448
20-Oct	78	163	130	70	130	103	2168	1895	2413	1378	1170	980
27-Oct	93	60	80	30	70	120	1748	2040	2623	1293	783	923
**2023**	**leafthoppers**						**thrips**					
18-Aug	73	148	188	83	143	198	218	268	333	105	133	165
25-Aug	208	240	163	163	115	220	508	378	593	278	208	308
2-Sep	260	175	108	128	198	275	175	123	263	43	28	93
8-Sep	315	278	353	218	298	158	63	103	43	73	58	128
16-Sep	423	558	378	293	235	358	188	168	228	83	123	138
22-Sep	388	448	315	218	323	408	413	323	508	283	368	450
30-Sep	133	163	208	160	98	218	268	293	318	255	218	298
7-Oct	203	273	353	175	118	223	158	193	228	113	93	135
13-Oct	98	110	75	48	125	95	48	83	128	63	30	38
20-Oct	15	50	35	15	30	73	128	63	233	35	55	25
27-Oct	0	0	0	0	0	0	0	0	0	0	0	0
**2023**	**Mirid bug *Cyrtorhinus lividipennis***											
18-Aug	73	18	13	18	58	33						
25-Aug	30	38	93	80	50	133						
2-Sep	70	40	20	193	160	143						
8-Sep	90	113	68	153	123	98						
16-Sep	108	143	90	385	238	205						
22-Sep	118	65	78	228	203	163						

30-Sep	148	213	163	393	258	298						
7-Oct	163	118	203	245	223	173						
13-Oct	95	178	143	228	353	438						
20-Oct	108	148	218	208	283	163						
27-Oct	73	175	208	183	153	68						
**2024**	**white-backed planthopper**						**brown planthopper**					
29-Jul	78	60	25	63	93	45	0	0	0	0	0	0
5-Aug	183	138	93	38	73	120	53	18	13	0	35	13
12-Aug	113	153	228	133	148	213	238	73	138	53	88	168
19-Aug	638	533	828	413	283	218	708	563	850	385	548	293
26-Aug	478	293	623	288	218	375	763	493	893	428	363	273
2-Sep	345	453	403	223	180	258	568	463	718	173	285	143
9-Sep	623	423	678	368	303	433	733	478	810	238	370	158
16-Sep	663	808	853	345	263	233	883	723	903	405	498	568
23-Sep	473	283	213	163	115	178	1153	958	1243	498	783	558
30-Sep	188	273	243	223	168	138	948	718	803	433	368	333
8-Oct	228	270	363	183	138	198	360	118	573	228	290	438
14-Oct	90	40	128	98	40	158	303	143	348	133	310	198
**2024**	**leafthoppers**						**thrips**					
29-Jul	15	58	103	48	80	115	120	188	268	278	313	168
5-Aug	120	153	85	93	150	223	468	278	388	235	428	318
12-Aug	138	193	98	203	63	128	373	528	678	393	465	533
19-Aug	288	385	183	213	178	248	143	88	218	128	190	245
26-Aug	185	275	228	203	168	248	193	138	268	108	158	225
2-Sep	168	68	238	65	98	133	168	123	65	53	148	98
9-Sep	135	248	198	148	130	178	208	278	160	228	168	118
16-Sep	228	213	250	115	158	208	308	373	448	383	283	478
23-Sep	88	128	168	73	103	35	278	405	538	355	438	278
30-Sep	35	78	95	115	133	78	195	258	298	148	195	248
8-Oct	25	53	10	20	68	88	68	128	183	68	58	90
14-Oct	18	38	65	0	20	25	15	20	38	33	13	20
**2024**	**Mirid bug *Cyrtorhinus lividipennis***											
29-Jul	0	0	0	0	0	0						
5-Aug	70	13	25	68	0	23						
12-Aug	18	83	43	18	48	98						
19-Aug	23	73	33	148	108	68						
26-Aug	85	38	118	190	258	128						
2-Sep	98	168	123	208	325	408						
9-Sep	148	188	118	133	215	295						
16-Sep	95	43	98	78	133	188						
23-Sep	28	98	68	123	153	228						
30-Sep	110	148	58	218	258	313						
8-Oct	133	183	108	133	183	273						

14-Oct	108	88	48	93	103	123						
